# Acupuncture for comorbid mild-moderate depression and chronic musculoskeletal pain: study protocol for a randomized controlled trial

**DOI:** 10.1186/s13063-021-05260-2

**Published:** 2021-04-29

**Authors:** Sheng Li, Jing Liu, Jianpeng Huang, Ding Luo, Qian Wu, Baile Ning, Ling Chen, Jianhua Liu, Wen-Bin Fu

**Affiliations:** Department of Acupuncture and Moxibustion, the 2nd clinical hospital of Guangzhou University of Chinese Medicine, Guangzhou, Guangdong China

**Keywords:** Acupuncture, Depression, Chronic musculoskeletal pain, HAMD, BDNF

## Abstract

**Background:**

Depression and chronic musculoskeletal pain (CMSP) are the leading causes of years lived with disabling diseases worldwide. Moreover, they often commonly coexist, which makes diagnosis and treatment difficult. A safe and effective treatment is urgently needed. Previous studies have shown that acupuncture is a cost-effective treatment for simple depression or CMSP. However, there is limited evidence that acupuncture is effective for depression comorbid with CMSP.

**Methods:**

This is a randomized, sham acupuncture-controlled trial with three arms: real acupuncture (RA), sham acupuncture (SA), and healthy control (HC). Forty-eight depression combined CMSP participants and 12 healthy people will be recruited from GDTCM hospital and randomized 2:2:1 to the RA, SA, and HC groups. The patients will receive RA or SA intervention for 8 weeks, and HC will not receive any intervention. Upon completion of the intervention, there will be a 4-week follow-up. The primary outcome measures will be the severity of depression and pain, which will be assessed by the Hamilton Depression Rating Scale (HAMD-17) and Brief Pain Inventory (BPI), respectively. The secondary outcome measures will be cognitive function and quality of life, which will be measured by the Montreal Cognitive Assessment (MoCA), P300, and World Health Organization Quality of Life (WHOQOL-BREF). In addition, the correlation between brain-derived neurotrophic factor (BDNF) and symptoms will also be determined.

**Discussion:**

The aim of this study is to evaluate the clinical efficacy and underlying mechanism of acupuncture in depression comorbid with CMSP. This study could provide evidence for a convenient and cost-effective means of future prevention and treatment of combined depression and CMSP.

**Trial registration:**

Chinese Clinical Trial Registry ChiCTR1800014754. Preregistered on 2 February 2018. The study is currently recruiting.

## Background

Chronic musculoskeletal pain (CMSP) and depression are the leading causes of years lived with disabling disease worldwide [1]. Moreover, CMSP and depression are highly intertwined and could exacerbate one another’s symptoms [2, 3]. Unfortunately, the overlapping symptoms lead to poor physical functional outcomes, longer duration of symptoms, and lower treatment response. This places a profound burden on both individuals and society. Notably, this burden far exceeds social service capacity.

The high prevalence of depression and CMSP comorbidity may indicate a common pathogenesis. Both depression and chronic pain are associated with cognitive function [4]. An increasing number of studies have shown that people who suffer from chronic pain or depression have poor performance on cognitive function tests [5, 6]. Other studies found that cognitive function acts as a protective factor against the emergence of chronic pain. Brain-derived neurotrophic factor (BDNF) is a member of the neurotrophin family, which is crucial for the survival and neuroplasticity of neurons. Decreased BDNF levels and increased BDNF methylation status, especially in the promoter regions of exons I and IV, were identified in patients with depression [7, 8]. More interestingly, after antidepressive treatment, BDNF methylation decreased, and serum BDNF levels increased [9, 10]. Some studies have demonstrated that serum BDNF levels are tightly correlated with the course of depression [11]. Thus, some scientists consider BDNF and BDNF promoter methylation to be biomarkers for depression [10, 12–14].

Acupuncture has been widely used to improve chronic pain and depression [15–17]. However, research on depression comorbid with chronic pain is rare. A randomized, controlled trial of acupuncture or counseling compared with usual care for depression showed that acupuncture is a cost-effective treatment for depression patients with or without comorbid pain [18]. However, the evidence on the effect of acupuncture is not conclusive. Therefore, we propose to conduct a pilot RCT of acupuncture on comorbid mild-moderate depression and CMSP. The objective of this study is to (1) determine the clinical efficacy of acupuncture on comorbid mild-moderate depression and CMSP and (2) investigate the association between BDNF and comorbidity of depression and CMSP.

## Methods

### Study design

**Fig. 1:**
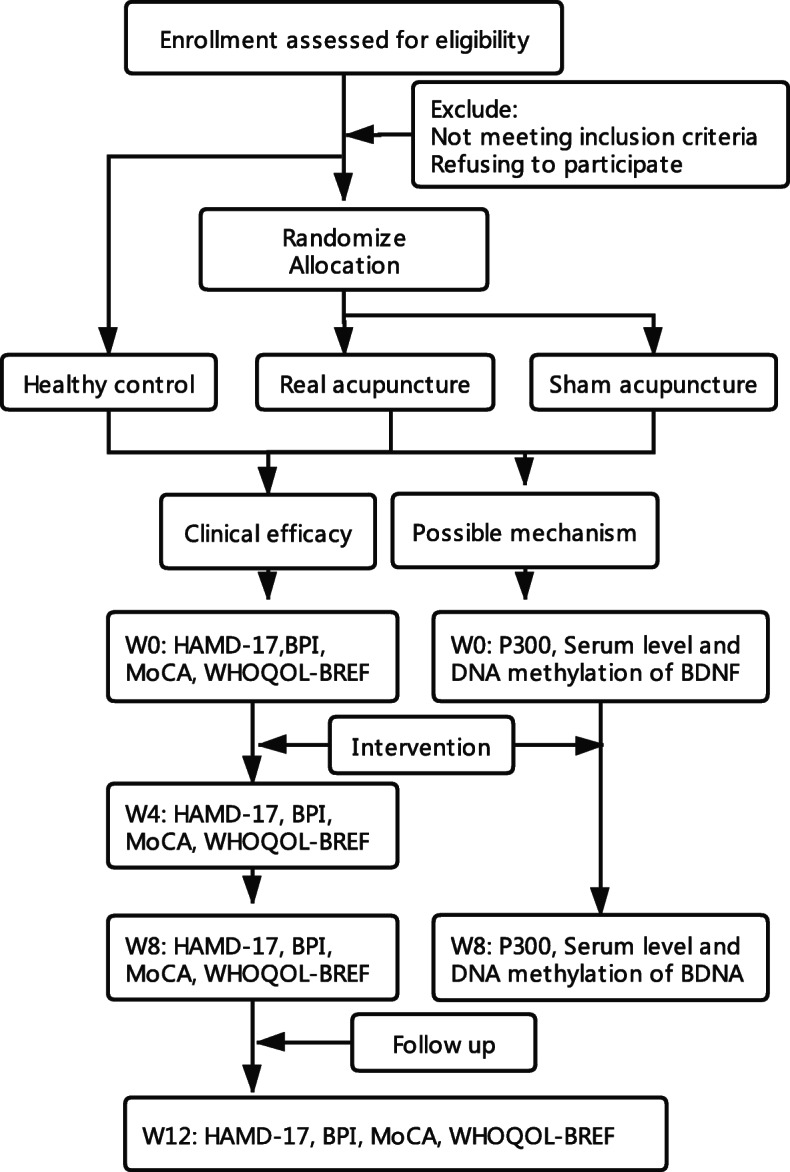
Flowchart of trial procedures

**Fig. 2:**
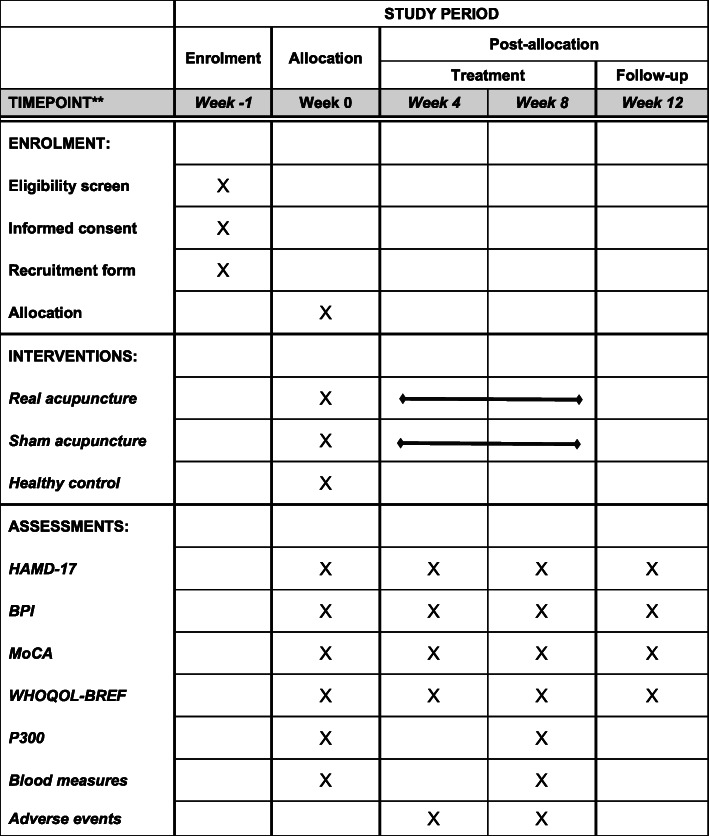
SPIRIT figure

This project is a randomized controlled clinical trial with 3 groups: real acupuncture (RA), sham acupuncture (SA), and healthy control (HC) at a ratio of 2:2:1. This trial was approved by the ethics committee of Guangdong Provincial Hospital of Chinese Medicine (Z2017-162-01). Before randomization, written informed consent will be obtained from all participants prior to their involvement. Primary and secondary outcome measures will be assessed at baseline, 4, 8, and 12 weeks. Outcome assessors and statisticians will be blinded in this study. An overview of the trial is shown in Fig. 1. This protocol was reported according to the Standard Protocol Items: Recommendation for Interventional Trials (SPIRIT) statement requirements. The complete SPIRIT checklist is available in Additional file 1 and Fig. 2.

### Types of participants

In this trial, we will recruit mild-moderate depression comorbid CMSP patients and healthy participants. Healthy participants need to meet the following criteria: no history of depressive disorder, a score on the 17-item Hamilton Depression Rating Scale (HAMD-17) < 8; no chronic pain in the past 3 months; and no complications of severe systemic diseases such as diabetes mellitus, tumors, or other conditions considered unsuitable to participate in the trial. CMSP patients with mild-moderate depression need to meet the following criteria.

### Inclusion criteria

Participants will be included if they meet the following criteria:

1. Meet the criteria of DSM-5™ and ICD-10 for mild-moderate depression;
2. HAMD-17 ≤ 24 and ≥ 8;
3. Suffering chronic musculoskeletal pain (location at the neck, back, and limb) more than 3 months and VAS score ≥ 4;
4. Aged between 18 and 65;
5. No acupuncture therapy during the past 3 months before enrollment; and
6. Voluntarily participating in this trial with a written informed consent form.

### Exclusion criteria

Participants with the following conditions will be excluded:

1. Suicidal ideation or SCL-90 depression score > 26;
2. Bipolar disorder or schizophrenia;
3. Malignant pain caused by cancer pain syndrome;
4. Suffering severe pain, VAS ≥7;
5. Headache and visceral pain such as stomachache;
6. Pregnant or breastfeeding women;
7. Complications of severe systemic diseases such as diabetes mellitus, tumors, or others considered not suitable to participate in the trial; and
8. Had acupuncture treatment in the last 3 months.

### Sample size calculation

In this study, a priori analysis was used to calculate the sample size. The HAMD-17 and BPI scores were used to calculate the sample size, and a larger sample was used. The HC participants did not have depression and pain symptoms, so the HC group will not be scored on HAMD-17 and BPI. Two group statistics were calculated for the sample size, and a *T* test was applied. According to the two-sample *T* test with *α* = 0.05, 32 depression comorbid CMSP participants were required to achieve 80% power. Allowing for a dropout of 15% at the end treatment time point, the recruitment goal of depression comorbid CMSP participants was 48 subjects (24 per group). The HC participant count was calculated to be 12, with a ratio of 2:2:1, as previously described. Therefore, a total of 60 participants will be recruited.

### Recruitment of participants

This trial will take place at the outpatient department of acupuncture and moxibustion, Second Affiliated Hospital of Guangzhou University of Chinese Medicine, China. Outpatients will be recruited via posters in the hospital and via Internet advertisement.

### Randomization

All eligible depression comorbid CMSP participants will be randomly allocated to RA or SA, and the healthy participants will be directly allocated to HC. A simple randomization method will be used. SPSS 22.0 software will be used to create the randomization sequence. Then, random numbers will be placed in opaque sealed envelopes and assigned to the patients by JL.

### Blinding

The participants, outcome assessors, and statisticians will be blinded to treatment allocation. All participants will be informed that they have an equal opportunity for allocation to the RA group or SA group before enrollment. In addition, to reduce bias during the intervention period, participants will be treated separately to prevent communication. All treatment sessions will be performed by trained and experienced acupuncturists. The acupuncturist will not provide any clues about the allocation information to the participants, assessors, or statisticians.

### Patient and public involvement

Neither patients nor the public will be directly involved in the design of the study and/or involved in the conduct of the study. The intervention in the study will be free for the participants. We will disseminate the main results to all the participants in the form of an accessible newsletter. Patient advisors were not used in the conduct of this study.

### Intervention

The study intervention protocol was determined according to the classical principles of traditional Chinese medicine. All acupuncturists had at least 5 years of acupuncture experience. The participants will be asked not to take any other medications for pain or depression and, if they did, to document the pills and dosage. In addition, the HC participants will not receive any medical intervention, except blood and P300 tests at baseline.

### Real acupuncture treatment

Participants in the RA group will receive acupuncture at Baihui (DU20), Yintang (EX-HN3), Renzhong (DU27), Chenjiang (RN24), Hegu (LI4, bilateral), and Taichong (LR3, bilateral). After skin disinfection, sterile adhesive pads will be placed on the above points, and then needles (Hwato, Suzhou, China) will be inserted through the pad. At the DU20 and EX-HN3 points, needles will be obliquely inserted about 16 mm deep, and at the DU27, RN24, L14, and LR3 points, needles will be vertically inserted approximately 12 mm, 12 mm, 25 mm, and 25 mm deep, respectively. All these points will be stimulated manually until patients feel heaviness, soreness, distension, or numbness sensation (deqi), as reported in our previous study [15]. Each acupuncture session will be continued for 30 min and then the pads will be removed with a disinfection swab. The acupuncture treatment will be administered twice per week for a total of 16 sessions over a period of 8 weeks.

### Sham acupuncture treatment

**Fig. 3:**
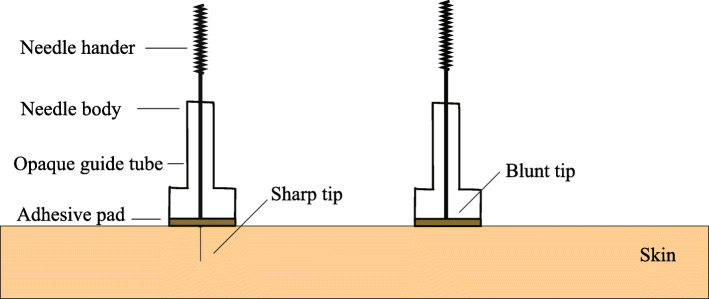
Illustration of the acupuncture needle structure

Participants in the SA group will receive the same procedure as the RA group but using non-insertion sham acupuncture [19] (Fig. 3). Briefly, after skin disinfection, sterile adhesive pads will be placed on the same acupoints as the RA group. Then, blunt needles will be inserted into the sterile adhesive pads but they will not break the skin and they will cause a tickling, pricking feeling similar to real acupuncture.

### Discontinuations

If participants are unable or unwilling to complete the treatment, we consider will them to be treatment dropouts. This could occur if participants withdraw consent to receive the study intervention or if the psychiatrist assessment suggests that it is inappropriate to continue the study intervention due to, for instance, depression or pain condition persists or getting worse, serious suicidal ideation, or a severe adverse event.

### Outcome measures

#### Primary outcomes

The primary outcomes are the changes in HAMD-17 and BPI scores from baseline to 12 weeks. HAMD-17 is a 17-item self-reported questionnaire; the higher the score is, the more severe the depression. A previous study showed that HAMD-17 has good validity and reliability for the assessment of depression severity. The BPI was first developed to measure pain in cancer patients, but it has become one of the most widely used pain measures, used for musculoskeletal pain and postsurgery pain [20, 21]. The BPI is a 15-item inventory that contains 2 dimensions: the intensity of the pain and interference with everyday activities. All the items are measured using a numerical rating scale from 0 to 10. The higher the score, the more severe the pain is.

#### Secondary outcomes

Secondary outcomes include the rates of depression response and remission at the 8th week (endpoint), where response is defined as a reduction of 50% of the HAMD-17 score and remission as a HAMD-17 score of 7 or less [22]; the rates of pain response at the 8th week, where response is defined as a reduction of 2 points of the BPI-WP [23, 24]; the Beijing version of Montreal Cognitive Assessment (MoCA); the World Health Organization Quality of Life BREF (WHOQOL-BREF); event-related potential (P300); and the serum level of BDNF and DNA methylation of BDNF. The Beijing version of the MoCA is highly recommended as a clinical and research tool for evaluating cognitive impairment. Previous studies proved that B-MoCA has a reliability of 0.73 Cronbach’s alpha in patients with obstructive sleep apnea-hypopnea syndrome with cognitive impairment [25]. The total MoCA score is 30 points, and less than 26 points is considered to indicate cognitive impairment. In addition, P300 will be used as an objective index to measure cognitive impairment, because it is based on the electrophysiological brain response. Previous studies have shown a decrease in amplitude and an increase in latency in depression patients [26]. The WHOQOL-BREF is an abbreviated version of the WHOQOL-100, which contains 26 items. In this trial, the official Chinese version of the WHOQOL-BREF, which has been approved by the WHOQOL Group [27], will be used to evaluate the changes in quality of life. BDNF is a biomarker for depression patients [14, 28]. All depression and CMSP participants will receive blood tests and P300 tests at baseline and 8 weeks. ELISA, RT-PCR, and BSP will be applied to measure the serum BDNA level and BDNF DNA methylation. The correlation between BDNF and symptoms will be determined.

### Incidence of adverse events

Acupuncturists will be asked to report and record any acupuncture-related adverse events (AEs), such as local bleeding, hematoma, fainting, local infection, unbearable prickling, or other discomfort after treatment. Severe AEs will be reported to the Research Ethics Committee within 24 h, which will provide medical advice to the research team, and the latter will evaluate whether the participant should continue the trial.

### Data management

All case report forms (CRFs) will be stored in a locked cabinet. The consent forms will be stored separately from the CRFs. Data from CRFs will be entered and locked by 2 data managers, and only authorized researchers will have access.

### Statistical analysis of the trial outcomes

Outcome data will be analyzed according to the intention-to-treat principle, including all randomly assigned participants after baseline assessment regardless of whether they receive the intervention. Missing data will be replaced by the data from the latest assessment. The outcome data will be analyzed with SPSS (version 22.0; SPSS Inc., Chicago, IL, USA). Descriptive statistics (variance analysis, non-parametric test, chi-square test) will be used to summarize demographic features among the three groups. The HAMD-17, BPI, MoCA, and WHOQOL-BREF will be analyzed using the repeated measures method, and post hoc comparisons will be evaluated with the Bonferroni test. The rates of depression/pain response and depression remission will be analyzed by the chi-square test. P300 and blood measures will be analyzed by paired *T* tests.

## Discussion

Depression and CMSP commonly coexist, which makes clinical treatment very difficult [29]. Despite huge costs, current pharmacological and psychological interventions have limited acceptability and effectiveness. Thus, patients are keen to have a cost-effective treatment. According to the American College of Physicians, acupuncture increases the response to second-generation antidepressants [30]. Previous studies also showed that acupuncture has clinical efficacy on depression and related symptoms, such as insomnia [15, 17, 18]. A systematic review showed that acupuncture is a cost-effective treatment for chronic pain [31]. Whether acupuncture has clinical efficacy on combined depression and CMSP is uncertain. On this basis, we designed this study, which meets CONSORT guidelines [32]. This trial will provide evidence on the clinical efficacy of acupuncture as a potential treatment for comorbid depression and CMSP. In addition, we will explore the mechanism of acupuncture’s antidepressant and analgesic effects from an epigenetic perspective.

Even so, there are some limitations of this study. First, it is a single-blind study, which may cause partial bias in the results. We will try our best to overcome this bias by allocating the different groups in different places to receive treatment. Second, the adherence of participants may be poor because the treatment period lasts 8 weeks. The study size is calculated on the hypothesis of a 15% loss rate, but it will be necessary to ascertain that there is no bias between the two intervention groups. Third, in this study, we performed a 4-week follow-up, and long-term follow-up will be more credible. Finally, there will be a skewed sex ratio in this study, because in traditional Chinese culture, men are generally unwilling to seek help, especially for mental disease. This bias was also present in our previous study.

We have presented the design and protocol for the clinical efficacy of acupuncture on mild-moderate depression comorbid with CMSP. On completion, this study will validate the clinical efficacy of acupuncture and its underlying mechanism.

## Supplementary Information

**Additional file 1.** Reporting checklist for protocol of a clinical trial.

## Data Availability

The authors declare that the data supporting the finds of this study are available within the article.

## Abbreviations

CMSP: Chronic musculoskeletal pain
BDNF: Brain-derived neurotrophic factor
RA: Real acupuncture
SA: Sham acupuncture
HC: Healthy control
MoCA: Montreal Cognitive Assessment
HAMD: Hamilton Depression Rating Scale
BPI: Brief Pain Inventory
CRF: Case report form
WHOQOL: World Health Organization Quality of Life
